# In Quest of the Missing C_2_H_6_O_2_ Isomers in the Interstellar Medium: A Theoretical Search

**DOI:** 10.1021/acs.jpca.4c04102

**Published:** 2024-08-01

**Authors:** Lisset Noriega, Luis Armando Gonzalez-Ortiz, Filiberto Ortíz-Chi, Sandra I. Ramírez, Gabriel Merino

**Affiliations:** † Departamento de Física Aplicada, Centro de Investigación y de Estudios Avanzados, Unidad Mérida, km 6 Antigua Carretera a Progreso, Apdo. Postal 73, Cordemex, 97310 Mérida, Yucatán, México; ‡ Centro de Investigaciones Químicas, Universidad Autónoma del Estado de Morelos, Av. Universidad 1001 Chamilpa, Cuernavaca, Morelos, C. P. 62209, México; § Conahcyt-Departamento de Física Aplicada, Centro de Investigación y de Estudios Avanzados del Instituto Politécnico Nacional, Mérida 97310, Yucatán, México

## Abstract

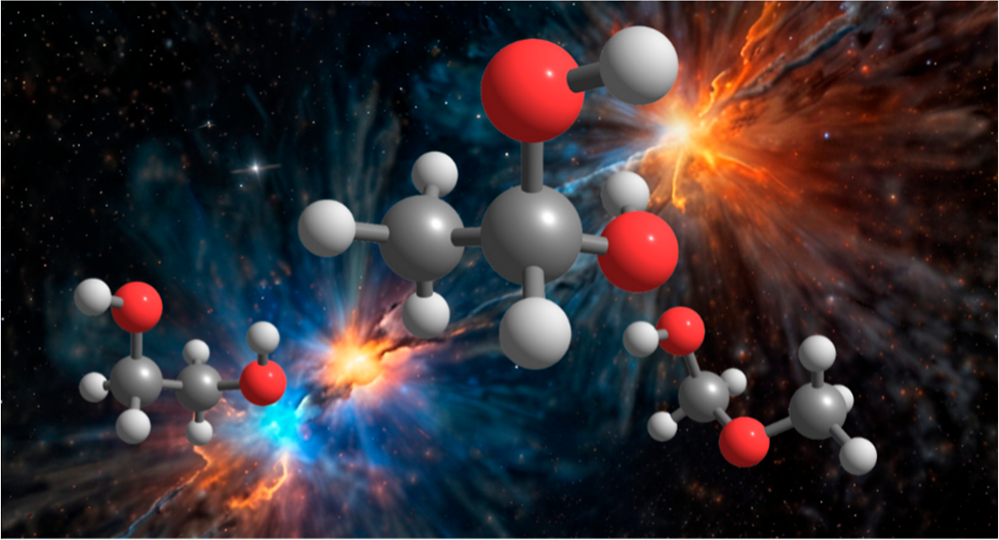

Ethylene glycol (C_2_H_6_O_2_), the only diol detected in the interstellar medium (ISM), is a key component in the synthesis of prebiotic sugars. Its structural isomer, methoxymethanol, has also been found in the ISM. Our results show that neither ethylene glycol (ethane-1,2-diol) nor methoxymethanol is the most stable isomer. Using high-level computational methods, we identified five isomers: two diols, one hydroxy ether, and two peroxides. The geminal diol 1,1-ethanediol (ethane-1,1-diol) is the most stable isomer, although it has not been detected in the ISM, whereas the two peroxides are less stable than the geminal diol by 60 kcal/mol. This study also provides the rotational constants and dipole moment for each conformer of every C_2_H_6_O_2_ isomer.

## Introduction

Observing organic molecules in interstellar space is a rapidly growing field within astrochemistry. Interstellar complex organic molecules (iCOMs) encompass various chemical entities, including alcohols, amines, carboxylic acids, aldehydes, and ketones. These iCOMs typically consist of six or more atoms,^1,2^ and certain iCOMs, including nucleobases, amino acids, and sugar derivatives, hold particular astrobiological interest.^3^ These organic compounds have been identified in meteorites such as the carbonaceous chondrites of Murchison (1969), Rennazo (1824), Murray (1950), and Ivuna (1938),^4,5^ suggesting that the essential building blocks of life might have arrived on early Earth via meteoritic impacts.^6−8^

One such iCOM, ethylene glycol (ethane-1,2-diol), HOCH_2_CH_2_OH, the simplest sugar alcohol, plays a crucial role in prebiotic sugar synthesis. It is the only diol confirmed in the interstellar medium (ISM)^9^ that has additionally been recovered from the Murchison and Murray meteorites.^10^ This diol can adopt various conformations, with the most stable one identified in different sources: the direction of the galactic central source Sgr B2(N-LMH),^11^ near the hot molecular core G31,410,31,^12^ or the high-mass star-forming region NGC 6334I.^13^ A higher-energy conformer was first identified in the protostar IRAS 16293-2422^14^ and later in the star-forming region Orion Kleinmann–Low nebula.^15^ Interestingly, another isomer of ethylene glycol, methoxymethanol, has been identified in the galactic protocluster region, the NGC 6334I region.^16^ These observations lead to two intriguing questions: First, why have only these two isomers with a composition of C_2_H_6_O_2_ been detected so far? Second, what factors influence the detection of specific conformers?

Rotational spectroscopy is highly effective for detecting iCOMs. However, for a molecule to be detectable with this technique, it must be rotationally active. This requires a nonzero dipole moment as the intensity of rotational lines depends on the square of this value. In 2020, Ellinger et al. proposed some criteria for the successful detection of molecules in the ISM, emphasizing factors such as (1) molecular rigidity, (2) a dipole moment around 2 D for enhanced detectability, (3) energy levels within 30 kcal/mol of the most stable isomer, and (4) weak adsorption on icy surfaces.^17^ With this in mind, we systematically explored the potential energy surface of the C_2_H_6_O_2_ stoichiometry to identify all their structural isomers. Our computations reveal that neither ethylene glycol nor methoxymethanol is the most stable system on the corresponding potential energy surface. Instead, 1,1-ethanediol (ethane-1,1-diol) is energetically more favorable than ethylene glycol and methoxymethanol by 11.7 and 15.7 kcal/mol, respectively. In fact, there are five different structural isomers of C_2_H_6_O_2_. Each structural isomer possesses several conformers, all with potential for detection, depending on their dipole moment. Consequently, we also conducted a global conformational search for each of the structural isomers. For each conformer, we computed its dipole moment and rotational constants, providing essential identification criteria.

## Methodology

Manually drawing all potential constitutional or structural isomers of small and saturated molecules on paper remains feasible but becomes impractical for larger and more complex molecules. To address this challenge, various methods have been developed for systematically enumerating all possible isomers by using combinatory restrictions. One such method uses the SMILES notation. SMILES provides a concise and machine-readable way to represent chemical structures using ASCII strings, offering a structured approach for detailing molecular constitutions. We recognize that our case study involving C_2_H_6_O_2_ is relatively straightforward. However, we are pursuing this approach because we are interested in exploring other iCOMs, and studying C_2_H_6_O_2_ isomers is a good starting point to test this approach.

Determining the number of conformers in a saturated organic molecule requires the number of dihedral angles (3^*n*^, where *n* is the number of dihedral angles). This process becomes simpler for molecules containing methyl groups. Initial analysis identified 47 conformers for the C_2_H_6_O_2_ system. However, after full geometry optimization, this number was reduced to 21. The decrease is attributed to two factors: (1) some initial structures converged to different local minima, and (2) certain conformers were identified as mirror images of others (Table S1). The conformational exploration for each structural isomer was performed using the Global Optimization of Molecular Systems (GLOMOS) software.^18^ GLOMOS implements a stochastic search algorithm to find the lowest-energy conformers from a starting structure with torsional degrees of freedom.

For all C_2_H_6_O_2_ isomers, full geometry optimizations were performed using the M06-2X-D3^19^/aug-cc-pVTZ^20^ level, including dispersion correction via Grimme’s D3 approximation.^21^ The resulting geometries served as the starting point for further reminimizations at the MP2^22^ level of theory with the same basis set. Harmonic vibrational frequency calculations were then performed on each optimized geometry at the same level. Finally, single-point calculations were achieved for energy refinement at the CCSD(T)/aug-cc-pVTZ//MP2/aug-cc-pVTZ level of theory. All computations were carried out using the Gaussian 16 package.^23^

## Results and Discussion

**Figure 1:**
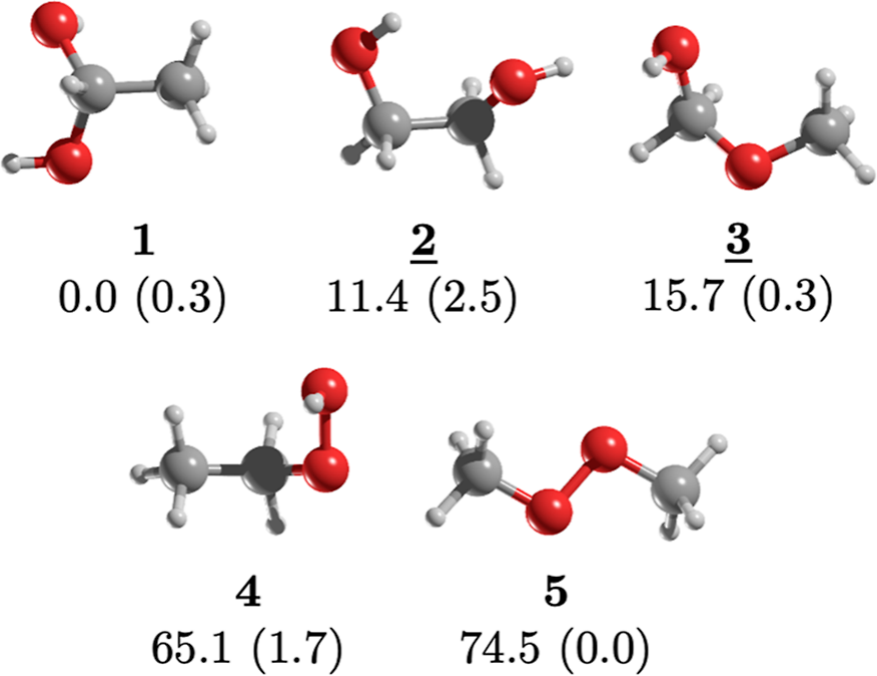
The most stable structural isomers of C_2_H_6_O_2_. Relative energy (in kcal/mol) obtained at the CCSD(T)/aug-cc-pVTZ//MP2/aug-cc-pVTZ level of theory. The dipole moment (in Debyes) is in parentheses, and the underlined isomers are those already detected in the ISM.

We found five distinct structural isomers of C_2_H_6_O_2_ (see Figure 1). Surprisingly, the most stable isomer is not ethylene glycol (**2**) but rather the geminal diol, 1,1-ethanediol (**1**). Notably, **1** is 11.4 kcal/mol more stable than **2** at the CCSD(T)/aug-cc-pVTZ//MP2/aug-cc-pVTZ level of theory. The hydroxy ether, methoxymethanol (**3**), ranks third in stability, being 15.7 kcal/mol less stable than **1**. The two peroxides, ethyl hydroperoxide—hydroperoxyethane— (**4**) and dimethyl peroxide—methylperoxymethane— (**5**), are significantly higher in energy than **1** by 65.1 and 74.5 kcal/mol, respectively. While prioritizing, descriptions based only on stability (highest to lowest) might seem logical. Nevertheless, there are important detection-related details to consider. So, we will begin our analysis with methoxymethanol.

**Figure 2:**
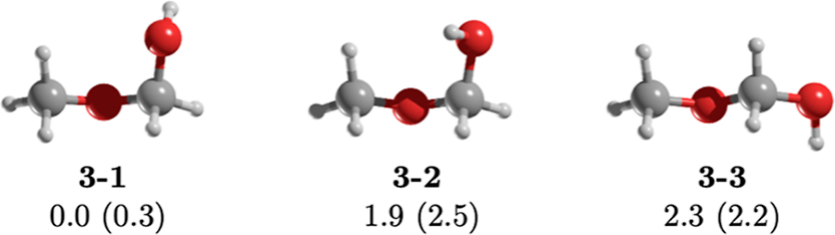
MP2/aug-cc-pVTZ geometries of the conformers of methoxymethanol, **3**. Relative energy (in kcal/mol) obtained at the CCSD(T)/aug-cc-pVTZ//MP2/aug-cc-pVTZ level of theory. The value in parentheses is the dipole moment in Debye.

Methoxymethanol is a hydroxy ether with three different conformers (Figure 2). The most stable one, **3–1**, boasts a dipole moment of 0.3 D and adopts a gauche conformation with a torsional angle OCOC of −67.5°. The rotation of the OH group alters the lone pair position, resulting in conformers with higher dipole moments. **3–2**, with a dipole moment of 2.5 D, is 1.9 kcal/mol less stable than **3–1**, while **3–3** (Δ*E* = 2.3 kcal/mol) has a *trans* arrangement and a dipole moment of 2.2 D (see Table 1).

**Table 1:** Relative Energies (Δ*E*, in kcal/mol) and Dipole Moment (μ, in Debye) of the Conformers of Each Structural Isomer of C_2_H_6_O_2_

isomer	Δ*E*^a^	μ^b^	detected in the ISM
**1** (1,1-Ethanediol)			
**1–1**	0.0	0.3	no
**1–2**	2.2	2.5	no
**1–3**	2.4	2.3	no
**1–4**	2.7	2.8	no
**1–5**	3.0	2.7	no
**2** (Ethylene Glycol)			
**2–1**	11.4	2.5	yes
**2–2**	11.8	2.5	yes
**2–3**	12.0	0.2	no
**2–4**	13.6	0.0	no
**2–5**	13.7	2.1	no
**2–6**	13.8	0.0	no
**2–7**	13.9	2.4	no
**2–8**	14.0	1.5	no
**2–9**	14.5	3.2	no
**3** (Methoxymethanol)			
**3–1**	15.7	0.3	yes
**3–2**	17.6	2.5	no
**3–3**	18.0	2.2	no
**4** (Ethyl Hydroperoxide)			
**4–1**	65.1	1.7	no
**4–2**	65.3	1.6	no
**4–3**	65.3	1.8	no
**5** (Dimethyl Peroxide)			
**5–1**	74.5	0.0	no

a CCSDT/aug-cc-pVTZ//MP2/aug-cc-pVTZ.

b MP2/aug-cc-pVTZ.

Small changes in the molecular geometry can significantly impact spectroscopic parameters such as rotational constants and dipole moments. Since these parameters define the rotational spectrum, rotational spectroscopy is highly effective at distinguishing even subtle variations in a molecule’s conformation. Therefore, obtaining accurate molecular geometry is crucial for identifying specific molecules and their dominant conformations in ISM. Motiyenko et al.^24^ synthesized and recorded the rotational spectra of **3–1** and **3–3** using a fast-scan terahertz spectrometer. Their experiments, conducted at 223 K and frequencies between 150 and 450 GHz, averaged the spectrum eight times. McGuire et al.^16^ used this data to identify **3–1** in the ISM and improve the root-mean-square (RMS) deviation from previously reported 40 kHz lines. **3–1** was detected in the MM1B source of NGC 6334I, known for its high dust temperatures averaging 154 K, and in IRAS 16293-2422 B,^25^ a region harboring hot corinos exceeding 100 K. At these and lower temperatures, the other conformers (**3–2** and **3–3**) were absent, making **3–1** the dominant form. These findings suggest that the requirement of a dipole moment greater than 2 D proposed by Ellinger et al. might be not essential, although a higher dipole moment can facilitate detection. The crucial factor seems to be the molecule’s energetic stability. With this in mind, we analyzed the rest of the structural isomers.

**Figure 3:**
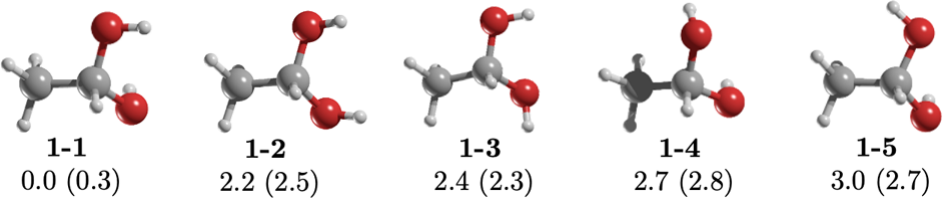
MP2/aug-cc-pVTZ geometries of the conformers of 1,1-ethanediol, **1**. Relative energy (in kcal/mol) obtained at the CCSD(T)/aug-cc-pVTZ//MP2/aug-cc-pVTZ level of theory. The value in parentheses is the dipole moment in Debye.

Despite being the most stable structural isomer of C_2_H_6_O_2_, 1,1-ethanediol (**1**) remains undetected in interstellar space. This can be attributed to the low dipole moment (only 0.3 D) of its most stable conformer, **1–1**. However, drawing parallels with **3**, the possibility for **1** detection in the ISM persists. **1** has four additional conformers (see Figure 3) where OH group rotation alters the lone pair positions, leading to conformers with higher dipole moments. All four remaining forms (**1–2** to **1–5**), with relative energies ranging from 2.2 to 3.0 kcal/mol, have dipole moments greater than 2.0 D. The current uncertainty revolves around whether the relative energy between these conformers is small enough to enable detection, considering typical ISM temperatures ranging from 10 to 100 K, with hotter regions found in hot molecular cores.^3^ Thus, determining the relative population of these conformers at such temperatures using the Boltzmann distribution equation becomes crucial. At temperatures below 100 K, the relative probability of these four conformers is practically negligible (Table S2). Consequently, if **1** is detected, then the dominant conformer would likely be **1–1**. Furthermore, free geminal diols are one of the most elusive classes of reactive intermediates. This presents challenges in obtaining pure samples and their rotational spectra. Recently, Kaiser and co-workers^26^ successfully prepared and characterized methanediol by processing low-temperature ice followed by sublimation into the gas phase. This approach offers a promising protocol for synthesizing and characterizing other unstable geminal diols. To aid future studies, we provide the rotational constants for 1,1-ethanediol and all its structural isomers in the Supporting Information (Table S3). Intriguingly, Kumar and Francisco^27^ proposed that **1** might be detectable not as a monomer but as a dimer stabilized by hydrogen bonding. These dimers possess suitable dipole moments and exhibit enhanced stability due to 7–11 kcal/mol hydrogen bonding energy.

**Figure 4:**
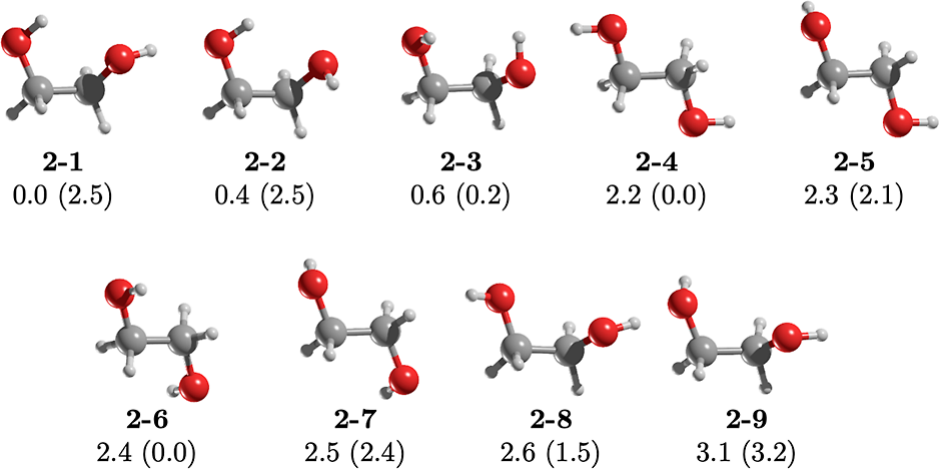
MP2/aug-cc-pVTZ geometries of the conformers of ethylene glycol, **2**. Relative energy (in kcal/mol) obtained at the CCSD(T)/aug-cc-pVTZ//MP2/aug-cc-pVTZ level of theory. The value in parentheses is the dipole moment in Debye.

Ethylene glycol, a familiar molecule encountered in various contexts,^28−31^ has nine conformers. The two most stable ones adopt a gauche conformation with torsional angles OCCO of 111.3 and 57.3°, respectively. One OH group forms an intramolecular hydrogen bond, while the other adopts either a *trans* (**2–1**) or a gauche (**2–2**) orientation relative to the C–C bond (Figure 4). The relative energy between these two forms is negligible at only 0.4 kcal/mol. At a temperature of 100 K, the relative populations of **2–1** and **2–2** are 84.6 and 11.3%, respectively. Both conformers have dipole moments of 2.5 D, explaining their successful detection within the ISM. Isomers **2–3** to **2–9** span an energy range of 0.6 to 3.1 kcal/mol. At 150 K, **2–3** has a relative population of 8.5%, although its dipole moment is a mere 0.2 D. Among the remaining six rotamers, four—**2–5** (2.1 D), **2–7** (2.4 D), **2–8** (1.5 D), and **2–9** (3.2 D)—possess dipole moments suitable for interstellar space detection. However, their relative populations become negligible at temperatures below 150 K. Consequently, only the two most stable ethylene glycol conformers can be observed within the ISM.

**Figure 5:**
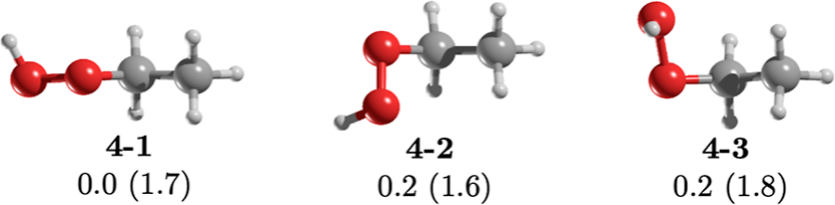
MP2/aug-cc-pVTZ geometries of the conformers of ethyl hydroperoxide, **4**. Relative energy (in kcal/mol) obtained at the CCSD(T)/aug-cc-pVTZ//MP2/aug-cc-pVTZ level of theory. The value in parentheses is the dipole moment in Debye.

Ethyl hydroperoxide (**4**) is an organic peroxide (R–O–OH) that acts as an intermediate in hydrocarbon oxidation.^32^ We identified three distinct conformers for **4** (see Figure 5). **4–1** and **4–3** correspond to previously studied *trans* and gauche conformations, respectively.^33−35^ Specifically, **4–1** has an OOCC dihedral angle of −176.7°, while **4–3** possesses a dihedral angle of 71.0°. To the best of our knowledge, **4–2** remains unexplored theoretically or experimentally. This conformer, with an OOCC dihedral angle of −66.5°, could also be designated as a gauche (g’) conformer. Interestingly, **4–2** and **4–3** have similar rotational constants but exhibit distinct dipole moment components, resulting in values of 1.6 and 1.8 D for **4–2** and **4–3**, respectively. Beyond hydrogen peroxide, detected in the ISM in 2011,^36^ reports on more complex peroxides within the ISM are null. All conformers of **4** have sufficiently strong dipole moments for detection via rotational spectroscopy. At 10 K, **4–1** dominates the population with 78.9%, while **4–2** and **4–3** contribute 10.5% each, suggesting their potential for ISM detection. However, the significant energy difference between **4** and **1** presents a challenge for their identification alongside **1**.

The final isomer is dimethyl peroxide (**5**), a molecule that has ignited significant debate regarding its most stable conformation. Historically, two potential structures contended for this title: a skewed conformation characterized by a COOC dihedral angle (Φ) of 120.0° and a *trans* conformation with a Φ of 180.0°.^37,38^ The *trans* conformation lacks rotational activity due to the absence of a dipole moment, unlike the skewed conformation. Prior experimental findings about the most stable structure were contradictory. One photoelectron spectroscopy study identified the *trans* conformation as the most stable.^39^ However, another investigation employing gas electron diffraction (GED) favored the skewed conformation as the lowest energy structure.^40^ Theoretical calculations added further complexity, revealing that the skewed conformation becomes less favorable as the basis set size increases.^41^ Our results initially considered both structures using DFT. However, subsequent optimization at the MP2 level with the aug-cc-pVTZ basis set identified only the *trans* structure, corroborating the findings of Ferchichi et al.^42^ This is in agreement with the photoelectron spectroscopy results, supporting the *trans* form as the most stable structure. Ferchichi et al. further reconciled the GED data by considering their fluxional character. This explanation definitively resolves the debate over the gas-phase structure of **5**, confirming the *trans* conformation as the most stable.^42^

## Conclusions

Our systematic exploration of the C_2_H_6_O_2_ potential energy surface yielded fascinating insights into the interplay between stability, dipole moment, and interstellar detectability. Despite having the most stable structure, 1,1-ethanediol’s low dipole moment hinders its detection via rotational spectroscopy. Fortunately, other techniques like rotovibrational spectroscopy offer alternative methods for observation. This method has proven successful in the ISM for molecules lacking a dipole moment, including the H_3_^+^ ion, tricarbon (C_3_), and benzene (C_6_H_6_). Similarly, methoxymethanol also exhibits a small dipole moment unfavorable for interstellar observation, yet it has been detected in the ISM. This suggests a possibility for 1,1-ethanediol detection as well. The significant energy difference between 1,1-ethanediol and ethyl hydroperoxide presents a hurdle for their detection despite its favorable dipole moments for rotational spectroscopy. However, the possibility of detecting a new peroxide in the ISM still exists. Therefore, we are convinced that this new automatized screening protocol based on SMILES opens the possibility of systematically understanding which other molecules could be potential candidates for detection in the ISM.

## Supporting Information Available

Number of conformers identified theoretically, by GLOMOS, and final number of conformers after optimization for each isomer of C_2_H_6_O_2_; Boltzmann distribution of the conformers of C_2_H_6_O_2_ at different temperatures at CCSD(T)/aug-cc-pVTZ//MP2/aug-cc-pVTZ; predicted equilibrium rotational constants, dipole moment components, and dipole moment at MP2/aug-cc-pVTZ of all conformers of C_2_H_6_O_2_ and their relative energy (Δ*E*, in kcal/mol) at CCSD(T)/aug-cc-pVTZ//MP2/aug-cc-pVTZ; and Cartesian coordinates of all the conformers identified (PDF)
