# Supplementary material for: In Quest of the Missing C2H6O2 Isomers in the Interstellar Medium: A Theoretical Search
Source: J Phys Chem A. 2024 Aug 1;128(32):6757–62. doi: 10.1021/acs.jpca.4c04102 (PMC11331521; doi:10.1021/acs.jpca.4c04102)
Supplement: Supplementary file 1 — jp4c04102_si_001.pdf [file jp4c04102_si_001.pdf]

SUPPLEMENTAL MATERIAL
FOR

In Quest of the Missing $C_2H_6O_2$ Isomers in the Interstellar Medium: A Theoretical Search

Lisset Noriega,[1] Luis Armando Gonzalez-Ortiz,[1] Filiberto Ortíz-Chi,[2] Sandra I. Ramírez,[3] and Gabriel Merino.[1,*]

[1] Departamento de Física Aplicada, Centro de Investigación y de Estudios Avanzados, Unidad Mérida, km 6 Antigua Carretera a Progreso, Apdo. Postal 73, Cordemex 97310, Mérida, Yucatán, Mexico.

[2] Conahcyt-Departamento de Física Aplicada, Centro de Investigación y de Estudios Avanzados del Instituto Politécnico Nacional, Mérida 97310, Yucatán, México

[3] Centro de Investigaciones Químicas, Universidad Autonóma del Estado de Morelos, Av. Universidad 1001 Chamilpa, Cuernavaca, Morelos, C. P. 62209, México

Email: gmerino@cinvestav.mx

lisset.noriega@cinvestav.mx

Table of content

Table S1. Number of conformers identified theoretically (#conf), identified using GLOMOS (initial #conf , and final number of conformers after optimization (Final # conf) for each isomer of $C_2H_6O_2$ (**A**).

Table S2. Boltzmann distribution of the conformers of $C_2H_6O_2$ at different temperatures (in Kelvin) considering the image mirror conformers and the relative energy at the CCSD(T)/aug-cc-pVTZ//MP2/aug-cc-pVTZ.

Table S3. Predicted equilibrium rotational constants (A_e, B_e, C_e, in MHz), dipole moment components (μ_a, μ_b, μ_c, in Debye), and dipole moment (μ, in Debye) at MP2/aug-cc-pVTZ of all conformers of $C_2H_6O_2$ and their relative energy (ΔE, in kcal/mol) at CCSD(T)/aug-cc-pVTZ//MP2/aug-cc-pVTZ.

X. Cartesian coordinates of all the conformers identified. All the structures are optimized at MP2/aug-cc-pVTZ. The relative energy (ΔE, in kcal/mol) includes the zero-point energy correction.

Table S1. Number of conformers identified theoretically (#conf), identified using GLOMOS (initial #conf , and final number of conformers after optimization (Final # conf) for each isomer of $C_2H_6O_2$ (**A**).

Isomer	# conf	Initial# conf	Final # conf
1	9	9	5
2	27	18	9
3	9	8	3
4	9	9	3
5	3	3	1

Table S2. Boltzmann distribution of the conformers of $C_2H_6O_2$ at different temperatures (in Kelvin) considering the image mirror conformers and the relative energy at the CCSD(T)/aug-cc-pVTZ//MP2/aug-cc-pVTZ.

Conformer	298K	150K	140K	100K	50K	10K
1-1	0.960	0.999	1.000	1.000	1.000	1.000
1-2	0.012	0.000	0.000	0.000	0.000	0.000
1-3	0.017	0.000	0.000	0.000	0.000	0.000
1-4	0.005	0.000	0.000	0.000	0.000	0.000
1-5	0.006	0.000	0.000	0.000	0.000	0.000
2-1	0.514	0.716	0.739	0.846	0.980	1.000
2-2	0.262	0.187	0.175	0.113	0.017	0.000
2-3	0.187	0.096	0.085	0.041	0.002	0.000
2-4	0.006	0.000	0.000	0.000	0.000	0.000
2-5	0.011	0.000	0.000	0.000	0.000	0.000
2-6	0.004	0.000	0.000	0.000	0.000	0.000
2-7	0.008	0.000	0.000	0.000	0.000	0.000
2-8	0.006	0.000	0.000	0.000	0.000	0.000
2-9	0.003	0.000	0.000	0.000	0.000	0.000
3-1	0.942	0.998	0.999	1.000	1.000	1.000
3-2	0.038	0.002	0.001	0.000	0.000	0.000
3-3	0.019	0.000	0.000	0.000	0.000	0.000
4-1	0.412	0.494	0.506	0.578	0.789	1.000
4-2	0.294	0.253	0.247	0.211	0.105	0.000
4-3	0.294	0.253	0.247	0.211	0.105	0.000
5-1	1.000	1.000	1.000	1.000	1.000	1.000

Table S3. Predicted equilibrium rotational constants (A_e, B_e, C_e, in MHz), dipole moment components (μ_a, μ_b, μ_c, in Debye) and dipole moment (μ, in Debye) at MP2/aug-cc-pVTZ of all conformers of $C_2H_6O_2$ and their relative energy (ΔE, in kcal/mol) at CCSD(T)/aug-cc-pVTZ//MP2/aug-cc-pVTZ.

#	Add. label	ΔE	A_e	B_e	C_e	μ_a	μ_b	μ_c	μ
1-1	I	0	9303.10	8725.99	5126.57	0.22	0.08	-0.07	0.25
1-2	II	2.2	9139.83	8963.96	5093.17	-1.21	0	2.14	2.45
1-3	III	2.4	9475.27	8696.63	5108.33	0.01	1.27	1.94	2.32
1-4	IV	2.7	8948.01	8835.92	5102.88	2.22	0	-1.77	2.84
1-5	V	3.0	9340.11	8689.51	5122.8	2.47	-0.76	-0.58	2.65
2-1	*g'Gt*	11.4	15344.00	5671.45	4670.89	-2.23	1.03	-0.56	2.52
2-2	*g'Gg*	11.8	15154.47	5620.93	4648.24	-1.38	1.45	1.44	2.46
2-3	*g'Gg'*	12.0	15127.11	5548.3	4555.09	0	0.23	0	0.23
2-4	*tTt*	13.6	30496.63	4032.8	3728.66	0	0	0	0
2-5	*gTt*	13.7	30012.98	3970.29	3705.39	0.95	-1.57	1.07	2.12
2-6	*gTg'*	13.8	29568.58	3908.8	3676.41	0	0	0	0
2-7	*gTg'*	13.9	29528.74	3913.22	3679.81	0	0	2.42	2.42
2-8	*tGt*	14.0	16663.08	5134.68	4511.17	0	1.49	0	1.49
2-9	*gGt*	14.5	16358.84	5142.92	4473.85	1.27	2.18	2.03	3.24
3-1	I	15.7	17156.20	5623.79	4851.81	0.23	0.08	-0.13	0.27
3-2	II	17.6	17091.14	5669.41	4797.38	0.77	-1.22	2.01	2.47
3-3	III	18.0	32534.52	4366.69	4086.59	-1.48	0.98	1.26	2.17
4-1	t	65.1	30870.09	4197.15	3915.18	0.84	0.65	1.35	1.72
4-2	g’	65.3	15642.00	5610.38	4694.96	-0.4	0.57	1.4	1.56
4-3	g	65.3	15477.19	5569.63	4691.69	1.46	-0.84	-0.53	1.77
5-1	I	74.5	31110.91	4638.48	4255.54	0	0	0	0

Cartesian coordinates of all the conformers identified. All the structures are optimized at MP2/aug-cc-pVTZ. The relative energy (ΔE, in kcal/mol) includes the zero-point energy correction.

Isomer 1

Conformer 1-1

ΔE= 0.0000000000

C	1.396957000	-0.017307000	-0.083730000
C	-0.043547000	-0.001218000	0.364894000
O	-0.726372000	1.168570000	-0.032210000
O	-0.651526000	-1.156492000	-0.174774000
H	1.436829000	-0.010918000	-1.173410000
H	1.893047000	-0.917003000	0.273384000
H	1.913281000	0.858988000	0.300313000
H	-0.122202000	0.001932000	1.453613000
H	-0.644376000	1.230887000	-0.992335000
H	-1.573856000	-1.149358000	0.107324000

Conformer 1-2

ΔE= 1.7903856581

C	-1.396822000	-0.000019000	-0.061764000
C	0.052106000	-0.000001000	0.343547000
O	0.632709000	-1.174297000	-0.188458000
O	0.632669000	1.174318000	-0.188451000
H	-1.453654000	0.000028000	-1.148592000
H	-1.890267000	0.888880000	0.322623000
H	-1.890222000	-0.888978000	0.322545000
H	0.165443000	-0.000001000	1.432832000
H	1.507000000	-1.291926000	0.197597000
H	1.506978000	1.291946000	0.197565000

Conformer 1-3

ΔE= 2.1691221888

C	1.402298000	0.055625000	-0.095864000
C	-0.037525000	-0.004282000	0.348976000
O	-0.815794000	1.050026000	-0.210533000
O	-0.574723000	-1.203890000	-0.126923000
H	1.435032000	0.020386000	-1.182654000
H	1.955606000	-0.788700000	0.310971000
H	1.863626000	0.980001000	0.247926000
H	-0.115026000	0.045557000	1.442423000
H	-0.679409000	1.840716000	0.322802000
H	-1.524331000	-1.175103000	0.039511000

Conformer 1-4

ΔE= 2.5508962355

C	-1.377410000	-0.000017000	-0.073573000

C	0.071285000	-0.000000000	0.369054000
O	0.739166000	-1.172700000	-0.044168000
O	0.739137000	1.172718000	-0.044167000
H	-1.430663000	-0.000015000	-1.166008000
H	-1.886336000	0.887855000	0.294936000
H	-1.886313000	-0.887905000	0.294931000
H	0.156753000	-0.000001000	1.452988000
H	0.528474000	-1.332284000	-0.971530000
H	0.528411000	1.332318000	-0.971519000

Conformer 1-5

ΔE= 2.7348359540

C	-1.366633000	-0.230281000	-0.105881000
C	0.054377000	-0.013396000	0.374496000
O	0.919173000	-1.032075000	-0.038274000
O	0.583891000	1.211287000	-0.117520000
H	-1.389916000	-0.228360000	-1.196031000
H	-2.027587000	0.554909000	0.263561000
H	-1.733811000	-1.188450000	0.255125000
H	0.104152000	-0.029342000	1.465112000
H	1.026087000	-0.936875000	-0.993118000
H	-0.129905000	1.856486000	-0.159988000

Isomer 2

Conformer 3-1

ΔE= 0.0000000000

O	1.433311000	-0.568856000	-0.190868000
C	0.726426000	0.569595000	0.274980000
C	-0.679232000	0.603874000	-0.271930000
O	-1.323882000	-0.574331000	0.220019000
H	0.865852000	-1.326727000	-0.003537000
H	0.685822000	0.583689000	1.368365000
H	1.277710000	1.445349000	-0.063432000
H	-1.194587000	1.504665000	0.072593000
H	-0.647075000	0.598992000	-1.363610000
H	-2.146316000	-0.701282000	-0.261888000

Conformer 3-2

ΔE= 0.4770920895

O	1.436370000	-0.567014000	-0.161006000
C	0.721464000	0.572191000	0.286653000
C	-0.676036000	0.598585000	-0.291275000
O	-1.382895000	-0.596616000	0.052491000
H	0.821344000	-1.308735000	-0.093391000
H	0.667192000	0.590420000	1.382170000
H	1.279507000	1.449911000	-0.038568000
H	-1.214996000	1.489323000	0.041519000
H	-0.625492000	0.603359000	-1.378290000

H	-1.627924000	-0.539900000	0.982407000

Conformer 3-3

ΔE= 0.7533193314

O	-1.426611000	-0.549781000	0.203917000
C	-0.712114000	0.595772000	-0.254975000
C	0.712111000	0.595774000	0.254977000
O	1.426609000	-0.549777000	-0.203922000
H	-1.183734000	-1.283863000	-0.368920000
H	-0.713034000	0.639594000	-1.346357000
H	-1.242058000	1.467854000	0.127890000
H	1.242051000	1.467860000	-0.127885000
H	0.713032000	0.639590000	1.346359000
H	1.183779000	-1.283850000	0.368944000

Conformer 3-4

ΔE= 2.2458333582

O	1.773059000	0.260275000	0.000010000
C	0.567035000	-0.498635000	-0.000004000
C	-0.567035000	0.498635000	-0.000004000
O	-1.773059000	-0.260275000	0.000010000
H	2.514334000	-0.352068000	-0.000041000
H	0.490811000	-1.130979000	-0.887454000
H	0.490798000	-1.130991000	0.887437000
H	-0.490798000	1.130992000	0.887436000
H	-0.490811000	1.130978000	-0.887455000
H	-2.514334000	0.352068000	-0.000036000

Conformer 3-5

ΔE= 2.4757466987

O	1.835072000	0.133583000	-0.085825000
C	0.567732000	-0.508226000	0.001896000
C	-0.563120000	0.498984000	-0.043906000
O	-1.776072000	-0.241966000	0.046805000
H	2.003944000	0.577429000	0.751081000
H	0.498720000	-1.171949000	-0.857029000
H	0.481223000	-1.114184000	0.906561000
H	-0.465867000	1.199053000	0.792791000
H	-0.504200000	1.065011000	-0.976117000
H	-2.513490000	0.367152000	-0.053073000

Conformer 3-6

ΔE= 2.5594083200

O	-1.837242000	0.102482000	-0.115749000
C	-0.562964000	-0.506209000	0.063533000
C	0.562963000	0.506209000	-0.063534000
O	1.837242000	-0.102482000	0.115750000
H	-2.020909000	0.643770000	0.658646000
H	-0.488402000	-1.018180000	1.025606000
H	-0.477698000	-1.256288000	-0.722846000
H	0.488403000	1.018179000	-1.025607000

H	0.477698000	1.256288000	0.722845000
H	2.020912000	-0.643765000	-0.658648000

Conformer 3-7

ΔE= 2.7405943826

O	0.705659000	1.703262000	-0.063564000
C	0.705659000	0.281063000	-0.029261000
C	-0.705659000	-0.281063000	-0.029261000
O	-0.705659000	-1.703262000	-0.063564000
H	0.348686000	2.024501000	0.770743000
H	1.227447000	-0.047130000	-0.925382000
H	1.258057000	-0.095488000	0.838720000
H	-1.258057000	0.095488000	0.838720000
H	-1.227447000	0.047130000	-0.925382000
H	-0.348686000	-2.024501000	0.770743000

Conformer 3-8

ΔE= 2.5733923435

O	-1.431988000	-0.544194000	0.252821000
C	-0.685546000	0.529034000	-0.310562000
C	0.685546000	0.529034000	0.310562000
O	1.431988000	-0.544194000	-0.252821000
H	-2.185301000	-0.722999000	-0.317091000
H	-0.578957000	0.414109000	-1.391984000
H	-1.169481000	1.488235000	-0.102639000
H	1.169481000	1.488235000	0.102638000
H	0.578957000	0.414109000	1.391984000
H	2.185302000	-0.722999000	0.317091000

Conformer 3-9

ΔE= 3.1477330211

O	-1.489571000	-0.580064000	0.061434000
C	-0.694522000	0.536651000	-0.307972000
C	0.683703000	0.532638000	0.309906000
O	1.414580000	-0.563749000	-0.221072000
H	-1.586926000	-0.574002000	1.019184000
H	-0.598911000	0.485173000	-1.391077000
H	-1.189717000	1.478411000	-0.049249000
H	1.171135000	1.484724000	0.074089000
H	0.596579000	0.456076000	1.400110000
H	2.272683000	-0.595621000	0.212445000

Isomer 3

Conformer 3-1

ΔE= 0.0000000000

C	1.466633000	-0.415497000	0.105407000
O	0.578563000	0.580665000	-0.387320000
C	-0.659357000	0.551408000	0.263145000
O	-1.406912000	-0.610216000	-0.015440000
H	2.403431000	-0.292812000	-0.429304000

H	1.072562000	-1.416779000	-0.066590000
H	1.638188000	-0.277192000	1.176647000
H	-0.529754000	0.552308000	1.348042000
H	-1.180669000	1.450815000	-0.062814000
H	-1.620617000	-0.595402000	-0.955213000

Conformer 3-2

ΔE= 1.8951881489

C	-1.447837000	0.432646000	0.082326000
O	-0.601332000	-0.627308000	-0.324502000
C	0.668525000	-0.550355000	0.258059000
O	1.441978000	0.533924000	-0.203290000
H	-2.440566000	0.212584000	-0.298219000
H	-1.116600000	1.389078000	-0.327586000
H	-1.488605000	0.500693000	1.175114000
H	0.570671000	-0.506463000	1.349945000
H	1.188988000	-1.453049000	-0.044902000
H	1.236814000	1.310487000	0.325669000

Conformer 3-3

ΔE= 2.2175120422

C	-1.744051000	0.051797000	0.030550000
O	-0.453723000	-0.519528000	-0.086964000
C	0.542278000	0.473014000	0.016648000
O	1.794575000	-0.123783000	-0.070339000
H	-2.460793000	-0.759701000	-0.046948000
H	-1.930652000	0.774256000	-0.769664000
H	-1.869801000	0.554111000	0.994843000
H	0.473758000	1.175570000	-0.818160000
H	0.408594000	1.013109000	0.964579000
H	1.862707000	-0.759723000	0.650585000

Isomer 4

Conformer 4-1

ΔE= 0.0000000000

C	1.807304000	-0.150915000	0.004102000
C	0.452166000	0.520536000	0.022905000
O	-0.501523000	-0.534455000	-0.030871000
O	-1.803450000	0.115257000	-0.093251000
H	1.922723000	-0.810877000	0.861633000
H	2.588446000	0.606779000	0.042884000
H	1.933161000	-0.733444000	-0.906059000
H	0.303563000	1.104966000	0.933966000
H	0.313953000	1.172908000	-0.841071000
H	-2.178885000	-0.204469000	0.739581000

Conformer 4-2

ΔE= 0.3852078727

C	-1.475929000	-0.510882000	-0.096592000
C	-0.554295000	0.625128000	0.289399000

O	0.693954000	0.577274000	-0.395248000
O	1.367335000	-0.620524000	0.093720000
H	-1.649863000	-0.508026000	-1.170948000
H	-2.433051000	-0.401086000	0.413420000
H	-1.041261000	-1.466405000	0.186503000
H	-0.958070000	1.589673000	-0.022820000
H	-0.375895000	0.649282000	1.367013000
H	2.149172000	-0.202908000	0.482213000

Conformer 4-3

ΔE= 0.5188497074

C	1.460917000	-0.533778000	-0.095944000
C	0.565568000	0.633284000	0.259725000
O	-0.702185000	0.587972000	-0.388325000
O	-1.429755000	-0.505063000	0.244149000
H	1.031160000	-1.468652000	0.256432000
H	2.435925000	-0.409699000	0.375255000
H	1.603303000	-0.590289000	-1.174097000
H	0.409237000	0.706206000	1.337757000
H	0.980282000	1.576769000	-0.097970000
H	-1.563296000	-1.074642000	-0.526655000

Isomer 5

ΔE= 0.0000000000

C	-1.733961000	0.111104000	-0.000007000
O	-0.483643000	-0.550303000	0.000006000
O	0.483643000	0.550303000	0.000012000
C	1.733961000	-0.111104000	-0.000006000
H	-2.469131000	-0.690833000	-0.000007000
H	-1.847709000	0.724961000	-0.893742000
H	-1.847721000	0.724970000	0.893719000
H	1.847721000	-0.724978000	0.893715000
H	1.847710000	-0.724952000	-0.893746000
H	2.469131000	0.690833000	0.000002000
